# Pedestrian Traffic Deaths Among Residents, Visitors, and Homeless Persons — Clark County, Nevada, 2008–2011

**Published:** 2014-07-18

**Authors:** Kaci L. Hickox, Nancy Williams, Laurie F. Beck, Tom Coleman, John Fudenberg, Byron Robinson, John Middaugh

**Affiliations:** 1 EIS officer, CDC; 2 Southern Nevada Health District; 3 Division of Unintentional Injury Prevention, National Center for Injury Prevention and Control, CDC; 4 Clark County, Nevada Office of the Coroner/Medical Examiner; 5 Division of Scientific Education and Professional Development, Center for Surveillance, Epidemiology, and Laboratory Services, CDC

Motor vehicle collisions and crashes are a leading cause of death among Nevada residents aged 5–34 years, representing 14% of all injury deaths in that age group in 2010 (1). During 2008–2011, a total of 173 pedestrian deaths from motor vehicle collisions occurred in Nevada, accounting for 16% of motor vehicle deaths in the state (2). Approximately 75% (2 million persons) of Nevada residents live in Clark County, which includes the city of Las Vegas. To analyze pedestrian traffic deaths in Clark County among residents, visitors, and homeless persons, the Southern Nevada Health District used coroner’s office data and death certificate data for the period 2008–2011. The results indicated that the average annual pedestrian traffic death rates from motor vehicle collisions during this period were 1.4 per 100,000 population for residents, 1.1 for visitors, and 30.7 for homeless persons. Among the three groups, time of day, location of motor vehicle collisions, and pedestrian blood alcohol concentration (BAC) differed. Effective interventions to increase roadway safety, such as lowering speed limits in areas with greater pedestrian traffic, targeting interventions during hours when alcohol-impaired walking is more likely, and modifying roadway designs to increase protection of pedestrians, might decrease pedestrian deaths among all three groups.

Clark County death certificate data for 2008–2011 includes all deaths of Clark County residents and all deaths occurring in Clark County. All motor vehicle–related deaths, including pedestrian deaths, occurring in Clark County are investigated by the Clark County Office of the Coroner/Medical Examiner. A motor vehicle traffic–related pedestrian death was defined as the death of a nonoccupant (i.e., a person on foot, in a wheelchair, or on a skateboard) within 30 days of being struck by a motor vehicle in transit on a public trafficway in Clark County, Nevada. Pedestrian deaths were identified in coroner’s office data and death certificate data independently, and discrepancies were reconciled through case reviews. Demographic information was obtained from death certificate data. Residence status, collision location, and BACs were abstracted from coroner’s office case reports.

Annual resident pedestrian death rates per 100,000 population were calculated by using 2008–2011 Nevada state census data and analyzed by age group and race/ethnicity. Pedestrian death rates for visitors aged ≥20 years per 100,000 population were calculated by using 2008–2011 Las Vegas Convention and Visitors Authority profiles. Visitor person-year estimates were calculated by multiplying the estimated number of yearly visitors aged ≥20 years by the average number of days and nights stayed and dividing by 365. Homeless pedestrian death rates per 100,000 population per year were calculated by using 2009 and 2011 homeless census surveys that provided point-in-time estimates of both sheltered and unsheltered homeless persons in Clark County (3). Because the homeless census survey is only performed every other year, 2008–2011 denominators were calculated using the census for 2009 (13,338) as the denominator for 2008–2009 and the census for 2011 (9,432) as the census for 2010–2011 (3).

The death certificate race/ethnicity variable includes seven mutually exclusive categories: white, black, American Indian, Asian, Hispanic, other, and unknown. Geographic location of collision was abstracted from coroner’s office data in one of three formats: street address, interstate name and mile marker, or intersection of two roads. Collision sites were mapped for those collisions occurring within a 22-mile (35-km) radius of Las Vegas Boulevard and Flamingo Road (a main intersection centrally located in the county). Time of collision was categorized as midnight–5:59 a.m., 6:00 a.m.–11:59 a.m., noon–5:59 p.m., and 6:00 p.m.–11:59 p.m. BACs from coroner’s office data for those aged ≥16 years were categorized as zero, 0.01–0.07 g/dL, and ≥0.08 g/dL. Pedestrians with BACs of ≥0.08 g/dL were considered impaired.

During 2008–2011, a total of 140 pedestrian traffic deaths occurred in Clark County; decedents were 107 residents, 19 visitors, and 14 homeless persons (Table 1). Excluding one fetal death, the median age for decedents was 50 years (range = 6–93 years); 100 (71%) of the decedents were male. Annual rates of pedestrian deaths among residents, visitors, and homeless persons were 1.4, 1.1, and 30.7 per 100,000 population, respectively. Death rates increased with age among residents, and those aged ≥60 years had higher rates than those in younger age groups (Table 1). Age group–specific death rates could not be calculated for visitors or homeless persons because age-specific census data for these groups were not available. Among residents, death rates per 100,000 population were highest among blacks (2.8), followed by whites (1.4), Pacific Islanders (1.0), and Hispanics (0.9) (Table 1).

Fatal collision sites among resident pedestrians were distributed throughout the urban areas of the county (Figure). Among visitors, collision sites were concentrated near the tourist area referred to as “The Strip,” along Las Vegas Boulevard and Interstate 15. Among homeless persons, collision sites were concentrated in northeastern Las Vegas.

The greatest percentage of pedestrian deaths among residents and homeless persons occurred during 6 p.m.–11:59 p.m. (residents: 41.1%; homeless persons: 78.6%). Visitor pedestrian deaths were more equally distributed throughout the day, peaking during midnight–5:59 a.m. (36.8%) (Table 2). Among the 122 decedents aged ≥16 years with BACs recorded, BAC ranges were 0–0.37 g/dL for residents, 0–0.34 for visitors, and 0–0.40 for homeless persons (0–0.40 g/dL overall). Overall, 32.0% of the decedents had a BAC of ≥0.08 g/dL, indicating impairment (26.4% among residents, 64.3% among homeless persons, and 35.3% among visitors) (Table 2). The proportion of alcohol impaired pedestrians was significantly greater (p<0.01) among homeless pedestrians, compared with residents.

## Discussion

The findings in this report indicate differences in pedestrian traffic deaths among residents, visitors, and homeless persons in Clark County during 2008–2011. Among the three groups, homeless persons had much higher pedestrian death rates. Although coroner’s office data did not specify whether a homeless decedent was sheltered or unsheltered, 78.6% of homeless pedestrian deaths occurred after homeless shelter curfews (6 p.m.). Nevada’s proportion of unsheltered homeless persons (60%) is among the highest of any state and twice the national proportion of unsheltered homeless (30%) (3). Therefore, the unsheltered homeless population might be at greater risk for pedestrian death in Clark County.

Increases in resident pedestrian death rates with age are consistent with previous studies (4,5). Differences in age distribution among resident and visitor pedestrian deaths might result from differences in walking patterns, familiarity of streets and terrains, and alcohol consumption patterns. In this report, the finding that resident blacks had higher death rates, compared with whites and Hispanics, might be attributable to differences in walking patterns or neighborhood characteristics, but this has not been documented. Other studies have identified variation in pedestrian death rates among racial/ethnic groups (5). Geographic differences in the collision sites among residents, visitors, and homeless persons likely mirror different walking patterns among the three groups. Evidence-based interventions should be considered, including those that target driver and pedestrian behavior and those that provide dedicated infrastructure (e.g., refuge islands and raised medians) for pedestrians.

Similar to previous studies, approximately one third of all pedestrian deaths in this study were associated with alcohol-impaired walking; further implementation and evaluation of interventions targeting this group are needed (4,6). Pedestrians who have consumed alcohol are at greater risk for being fatally or seriously injured (7). Although research on effectiveness is limited, interventions that might reduce deaths related to pedestrian alcohol impairment include public awareness campaigns, along with legislative approaches (e.g., limiting availability of alcohol or prohibiting public intoxication), programs for early identification and treatment of persons with alcohol problems, and environmental interventions (e.g., improved lighting and speed control measures) (8). *The Guide to Community Preventive Services* recommends electronic screening and brief intervention. This intervention, which can be administered in almost any setting, involves use of electronic devices (i.e., computers, telephones, and mobile devices) to screen for excessive drinking and to deliver a brief intervention with personalized feedback about risks and consequences (9).

The World Health Organization’s pedestrian safety manual recommends six categories of pedestrian safety interventions: reducing pedestrian exposure to vehicular traffic, reducing vehicle speeds, improving the visibility of pedestrians, improving pedestrian and motorist safety awareness and behavior, and providing care for injured pedestrians (10). Proven interventions should be based on the needs in particular geographic locations and might include constructing pedestrian refuge islands and raised medians, upgrading traffic and pedestrian signals, constructing overpasses or underpasses, installing speed management measures, and developing and enforcing traffic laws (10).

The findings in this report are subject to at least four limitations. First, some visitor pedestrians might have been injured in Clark County but subsequently died elsewhere, although such events likely would be rare and have a negligible effect on the visitor pedestrian death rate. Second, the accuracy of visitor profiles and homeless census surveys has not been assessed, which might result in overestimation or underestimation of pedestrian death rates for those groups. Third, pedestrian death rates for visitors and homeless persons might be unstable because of the small numbers of deaths and fluctuations in the two populations. Finally, denominator data for residents are known to include some homeless persons; although an actual count could not be obtained, the number likely is small and unlikely to make a substantial difference in pedestrian death rates for residents.

What is already known on this topic?

Motor vehicle collisions and crashes are a leading cause of death for Nevada residents aged 5–34 years, representing 14% of all injury deaths in that age group in 2010. In Nevada, collisions with pedestrians accounted for 16% of motor vehicle deaths.

What is added by this report?

Average annual pedestrian death rates in Clark County, Nevada, during 2008–2011 were much higher among homeless persons (30.7 per 100,000 population), compared with residents (1.4) and visitors (1.1). Pedestrian deaths among these groups differed in the prevalence of alcohol involvement and the place and time of collision.

What are the implications for public health practice?

Using coroner’s office data to identify pedestrian deaths among homeless persons can help local health authorities identify high-risk geographic locations, times of collisions, and alcohol involvement to support intervention efforts. Because 32.0% of pedestrian deaths in Clark County involved alcohol impairment, interventions to decrease alcohol-impaired walking in areas with concentrations of alcohol-impaired pedestrians might reduce pedestrian deaths.

Higher pedestrian death rates among homeless persons in Clark County might be related to high rates of alcohol and drug abuse, mental illness, and increased time spent walking along roadways (3); further research of this population is needed to more fully understand behaviors and environments that increase their risk. Interventions to decrease pedestrian deaths among homeless persons can be enhanced by a deeper understanding of the association between homeless pedestrian deaths and excessive alcohol use, nighttime collisions, and not residing in shelters. Homeless service providers, especially those providing services to unsheltered homeless persons in Clark County, should understand that pedestrian death is a health risk for their clients and consider collaborating with organizations that have expertise in pedestrian safety to identify and implement effective interventions.

## Figures and Tables

**FIGURE:**
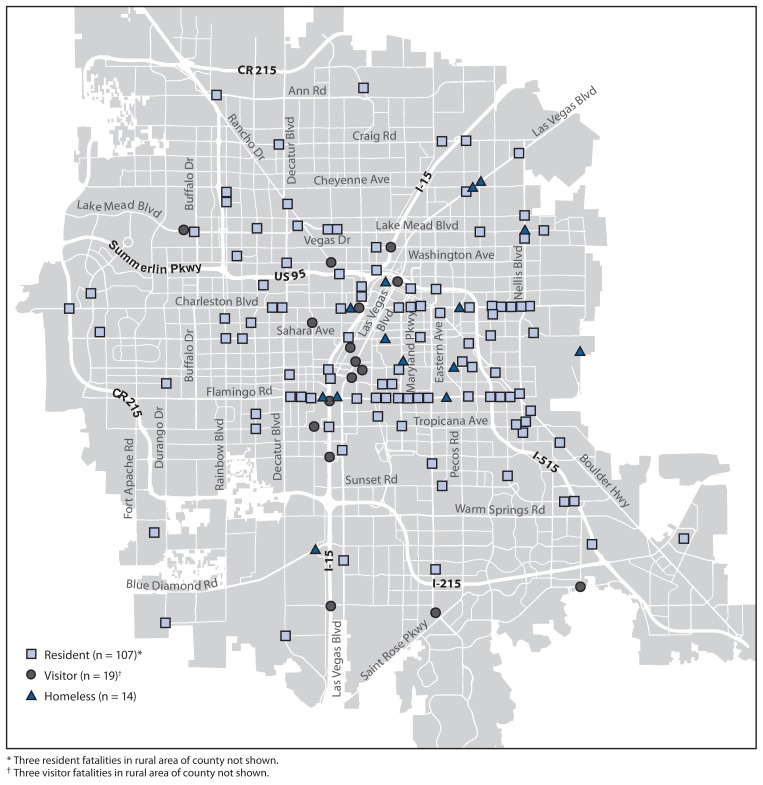
Pedestrian traffic deaths, by residence status — Clark County, Nevada, 2008–2011 * Three resident fatalities in rural area of county not shown. ^†^Three visitor fatalities in rural area of county not shown.

**TABLE 1:** Number of pedestrian traffic deaths and average annual death rates,* among residents, visitors, and homeless persons, by age group and race/ethnicity — Clark County, Nevada, 2008–2011

	Residents		Visitors		Homeless persons	
			
Characteristic	No.	Rate	No.	Rate	No.	Rate
**Overall pedestrian traffic deaths (N = 140)**	**107**	**1.4**	**19**	**1.1**	**14**	**30.7**
**Age group (yrs)**						
≤19	14	1.2	—†	—	—†	—
20–39	17	0.6	8	—	—†	—
40–59	38	0.9	8	—	11	—
60–79	30	1.5	—†	—	—†	—
≥80	7	2.2	—†	—	—†	—
**Race/Ethnicity** §						
White	60	1.4	11	—	10	—
Black	20	2.8	—†	—	—†	—
Asian	—†	—	—†	—	—†	—
Pacific Islander	6	1.0	—†	—	†	—
Hispanic	20	0.9	—†	—	—†	—

* Average annual death rates per 100,000 population for residents and homeless persons and average death rates per 100,000 person-years for visitors. Visitor overall death rate includes only persons aged ≥20 years.

† Subgroup values ≤5 were suppressed in accordance with state health department policy.

§ Persons categorized as white, black, Asian, and Pacific Islander were all non-Hispanic. Persons categorized as Hispanic might be of any race.

**TABLE 2:** Number and percentage of pedestrian traffic deaths among residents, visitors, and homeless persons, by time of day and blood alcohol concentration — Clark County, Nevada, 2008–2011

	Residents		Visitors		Homeless persons		Overall	
				
Characteristic	No.	(%)	No.	(%)	No.	(%)	No.	(%)
**Time of day**								
Midnight–5:59 a.m.	23	(21.5)	7	(36.8)	1	(7.1)	31	(22.1)
6:00 a.m.–11:59 a.m.	14	(13.1)	5	(26.3)	1	(7.1)	20	(14.3)
Noon–5:59 p.m.	26	(24.3)	3	(15.8)	1	(7.1)	30	(21.4)
6:00 p.m.–11:59 p.m.	44	(41.1)	4	(21.1)	11	(78.6)	59	(42.1)
**Total**	**107**	**(100.0)**	**19**	**(100.0)**	**14**	**(100.0)**	**140**	**(100.0)**
**Blood alcohol concentration (g/dL)** *								
Zero	61	(67.0)	10	(58.8)	4	(28.6)	75	(61.5)
0.01–0.07	6	(6.6)	1	(5.9)	1	(7.1)	8	(6.6)
≥0.08	24	(26.4)	6	(35.3)	9	(64.3)	39	(32.0)
**Total**	**91**	**(100.0)**	**17**	**(100.0)**	**14**	**(100.0)**	**122**	**(100.0)**

* A total of 18 deaths among persons aged <16 years or with missing blood alcohol concentration data were excluded. Pedestrians with blood alcohol concentration ≥0.08 g/dL were considered impaired.
